# Exploring the impact of a decision support algorithm to improve clinicians’ chemotherapy-induced peripheral neuropathy assessment and management practices: a two-phase, longitudinal study

**DOI:** 10.1186/s12885-021-07965-8

**Published:** 2021-03-06

**Authors:** Robert Knoerl, Emanuele Mazzola, Fangxin Hong, Elahe Salehi, Nadine McCleary, Jennifer Ligibel, Kaitlen Reyes, Donna L. Berry

**Affiliations:** 1 Dana-Farber Cancer Institute, Boston, MA USA; 2 Department of Biostatistics and Computational Biology, Dana-Farber Cancer Institute, Boston, MA USA; 3 Medical Oncology, Dana-Farber Cancer Institute, Boston, MA USA; 4 Biobehavioral Nursing and Health Informatics, University of Washington, Seattle, WA USA

**Keywords:** Peripheral nervous system diseases, Neoplasms, Algorithms, Decision Support systems, Clinical, Documentation, Chemotherapy-induced peripheral neuropathy

## Abstract

**Background:**

Chemotherapy-induced peripheral neuropathy (CIPN) negatively affects physical function and chemotherapy dosing, yet, clinicians infrequently document CIPN assessment and/or adhere to evidence-based CIPN management in practice. The primary aims of this two-phase, pre-posttest study were to explore the impact of a CIPN clinician decision support algorithm on clinicians’ frequency of CIPN assessment documentation and adherence to evidence-based management.

**Methods:**

One hundred sixty-two patients receiving neurotoxic chemotherapy (e.g., taxanes, platinums, or bortezomib) answered patient-reported outcome measures on CIPN severity and interference prior to three clinic visits at breast, gastrointestinal, or multiple myeloma outpatient clinics (*n* = 81 usual care phase [UCP], *n* = 81 algorithm phase [AP]). During the AP, study staff delivered a copy of the CIPN assessment and management algorithm to clinicians (*N* = 53) prior to each clinic visit. Changes in clinicians’ CIPN assessment documentation (i.e., index of numbness, tingling, and/or CIPN pain documentation) and adherence to evidence-based management at the third clinic visit were compared between the AP and UCP using Pearson’s chi-squared test.

**Results:**

Clinicians’ frequency of adherence to evidence-based CIPN management was higher in the AP (29/52 [56%]) than the UCP (20/46 [43%]), but the change was not statistically significant (*p* = 0.31). There were no improvements in clinicians’ CIPN assessment frequency during the AP (assessment index = 0.5440) in comparison to during the UCP (assessment index = 0.6468).

**Conclusions:**

Implementation of a clinician-decision support algorithm did not significantly improve clinicians’ CIPN assessment documentation or adherence to evidence-based management. Further research is needed to develop theory-based implementation interventions to bolster the frequency of CIPN assessment and use of evidence-based management strategies in practice.

**Trial registration:**

ClinicalTrials.Gov, NCT03514680. Registered 21 April 2018.

## Introduction

Chemotherapy-induced peripheral neuropathy (CIPN) is a frequent dose-limiting complication of neurotoxic chemotherapy (e.g., taxanes, oxaliplatin, bortezomib) [1, 2] associated with numbness, tingling, and pain in the hands or feet [2]. For example, taxane-induced peripheral neuropathy symptoms (e.g., numbness or tingling) generally present in a stocking-glove distribution [3], acute oxaliplatin-induced peripheral neuropathy symptoms are characterized by brief cold-induced neuropathic pain and muscle cramps [4], while bortezomib-induced peripheral neuropathy often presents with pain [5]. The symptoms of CIPN are difficult to assess and manage because patients have difficulty describing the sensations they are experiencing [6] and there are few treatment options [7]. Subsequently, poorly managed CIPN may impair physical function [8, 9] and increase health care utilization and expenditures [10].

To prevent the untoward effects of CIPN, clinicians should assess patients for CIPN regularly during neurotoxic chemotherapy to identify risk and manage early CIPN symptoms. However, qualitative and quantitative evidence suggests that CIPN is under assessed and managed by clinicians. Participants in qualitative studies have reported that clinicians do not adequately follow up on CIPN concerns or provide much feedback about how to manage CIPN [6, 11]. A medical record review of breast clinician’ notes by Knoerl et al. (2018) revealed that CIPN-related numbness or tingling was documented in approximately 28/48 notes (58.3%) [12]. Further, Knoerl et al. (2019) demonstrated that CIPN was discussed or documented in 44% and 46% of 159 audio recorded patient-clinician outpatient encounters and associated medical record notes (e.g., patients with breast, colorectal, prostate, and/or head and neck cancers), respectively [13]. In both studies, the first-line treatment recommended for painful CIPN (i.e., duloxetine) [7] was never prescribed to participants reporting CIPN [12, 13].

Previous research has demonstrated that the delivery of a web-based platform designed to capture CIPN patient-reported outcomes at the point of care and provide clinicians and patients with generated treatment and/or self-care information: 1) is feasible to implement in an outpatient oncology clinic [14], 2) improves clinician documentation of non-painful CIPN symptoms (i.e., numbness) [12], and 3) improves patients’ activation in their care [15]. However, the previously tested web-based platform was delivered in one breast oncology clinic setting and in a relatively small number of patients (*N* = 25 Phase I, and *N* = 75 Phase II) and clinicians (*N* = 6) [12]. To build upon this research, we revised the Algorithm for Nursing Assessment and Management of CIPN, an algorithm developed to guide nurses’ decision making in the assessment and management of CIPN in clinical practice [16]. The revised algorithm incorporates data from the administration of standardized patient reported outcome measures and objective assessments (e.g., reflex and vibration testing) to generate evidence-based CIPN management recommendations for clinicians based on the patients’ CIPN symptom presentation. Availability of the CIPN algorithm was hypothesized to influence clinicians’ CIPN assessment and management behaviors because the algorithm provided clinicians with CIPN assessment and evidence-based management strategies and CIPN patient-reported outcome scores for use during the clinical encounter.

### Purpose

The primary aims of this two-phase, pre-post longitudinal study were to explore the impact of a CIPN clinician decision support algorithm on clinicians’ CIPN assessment documentation and adherence to evidence-based management in comparison to usual care. Secondarily, we assessed changes in patients’ self-reported CIPN severity, worst pain intensity, and CIPN-related functional impairment between phases. Also, we determined the feasibility of algorithm delivery and clinicians’ ratings of acceptability and satisfaction with the algorithm.

## Methods

### Design, sample, setting

The study aims were conducted using a two-phase, pre and post-test design. One-hundred sixty-two patients (*n* = 81 for usual care phase [UCP] and *n* = 81 for algorithm phase [AP]) and 53 clinicians were recruited. Patients were eligible if they were 1) ≥ 18 years old, 2) finished at least one infusion of neurotoxic chemotherapy (e.g., taxanes, platinums, or bortezomib) for the treatment of breast, gastrointestinal or multiple myeloma malignancies, 3) scheduled to attend at least three more clinic visits associated with neurotoxic chemotherapy receipt after consent, 4) ambulatory, 5) proficient in English, and 6) receiving care from a clinician enrolled in the study. Patients were excluded if they had a prognosis of less than 2 months or documented peripheral neuropathy due to other causes (e.g., diabetes) [17, 18] prior to starting neurotoxic chemotherapy. Of note, the patient eligibility criteria were amended during the trial to enhance recruitment feasibility. At the onset of the trial, patients were eligible if they had received ≤1/3 of the total planned neurotoxic chemotherapy regimen at the time of consent, planned to receive neurotoxic chemotherapy for ≥ 3 months, and all three study visits could be completed within 12 weeks after consent. Clinicians were eligible if they were a medical doctor, nurse practitioner, or physician assistant providing care to patients at the breast, gastrointestinal, or multiple myeloma clinics at Dana-Farber Cancer Institute. Informed consent was obtained from all patient and clinician participants and study oversight and ethics approval was provided by the Dana-Farber/Harvard Cancer Center Office for Human Research Studies (18–049). Patients consented to either phase received a $30 Amazon gift card at the conclusion of the study.

### Measures

#### Feasibility, acceptability, and satisfaction

Feasibility of CIPN algorithm implementation was evaluated based on the number of times the clinicians received the CIPN algorithm prior to a clinic visit. In addition, a two-item feasibility questionnaire prompted clinicians to state how often they used the algorithm and other CIPN educational resources to guide the assessment and management of CIPN, respectively (each item scored from 1 to 5; 1 = “never,” 5 = ‘always’). Clinicians’ perspectives of acceptability and satisfaction with the CIPN algorithm were evaluated using an adapted subset of eight questions from the Adapted Acceptability E – Scale [19]. Questions pertained to clinicians’ ratings of satisfaction with algorithm use and how helpful the algorithm was in guiding patient interactions, promoting communication, and identifying areas of need (each item scored from 1 to 5; higher scores = greater acceptability and satisfaction). Only clinicians who received the algorithm during the AP were invited to complete the Feasibility Questionnaire and Adapted Acceptability E – Scale.

#### CIPN patient-reported outcomes

The Patient Reported Outcomes version of the Common Terminology Criteria for Adverse Events (PRO-CTCAE™) Numbness and Tingling Severity and Interference Items ask patients to self-report the severity of numbness and tingling in the hands or feet (“none [0],” “mild [1],” “moderate [2],” “severe [3],” or “very severe [4]”) at its worst in the past 7 days, and how much these symptoms have interfered with activities of daily living (“not at all [0]” to “very much [4]” interference) [20–22]. Strong evidence demonstrates the psychometric properties of the PRO-CTCAE™ Numbness and Tingling Severity and Interference Items [20–24]. A 0–10 numerical rating scale was used to measure participants’ worst CIPN pain intensity score over the past 7 days (0 = “No pain,” 10 = “Pain as bad as you can imagine”). Participants who reported PRO-CTCAE™ numbness and tingling severity or interference item scores ≥1/4 and/or 0–10 numerical rating scale of worst CIPN pain intensity scores ≥4/10 were prompted to complete additional questions asking about the location, duration, characteristics, and/or functional limitations associated with CIPN. The follow up screening questions were created by the study team using results from qualitative analyses published in the literature [6, 25]. Lastly, the QLQ-CIPN20 sensory (nine items) subscale measures patients’ self-reported severity of numbness, tingling, and pain in the hands/feet, while the motor subscale (eight items) measures patients’ self-reported functional limitations associated with CIPN symptoms (0–100 transformed score, higher scores = worse severity) [26]. Several studies support the psychometric properties of the QLQ-CIPN20 sensory and motor subscales [23, 27, 28].

#### Electronic medical record abstraction

Study staff abstracted clinicians’ documentation (yes/no) of CIPN assessment (i.e., numbness, tingling, pain, deep tendon reflexes, vibration sensibility, CIPN functional deficits, and functional motor assessments) and CIPN management (i.e., pharmacological or non-pharmacological treatment, continue to monitor, dose reduction, referral to subspecialty). Study staff also abstracted information about patients’ cancer treatment-related information, such as diagnosis, stage, chemotherapy type, pain medication use, comorbid conditions associated with increased CIPN risk (e.g., high body mass index [18, 29, 30] or diabetes [17, 18]), and prior neurotoxic chemotherapy exposure. All medical record abstraction was conducted by the principal investigator and two study staff members. All identified discrepancies were resolved between the principal investigator and study staff.

#### Criteria for appropriate CIPN management evaluation form

Several authors (RK, ES, DB) met to evaluate the appropriateness of clinicians’ CIPN management recommendations using a study-team created form. Appropriate clinician-related CIPN management was scored as yes or no (i.e., “yes” = appropriately managed CIPN given current evidence surrounding CIPN management). Clinicians were deemed to have provided appropriate management for moderate-severe CIPN (i.e., ≥ 2/4 on PRO-CTCAE™ Numbness and Tingling Severity Item) if a recommendation for pharmacological treatment (e.g., duloxetine, gabapentin) [31], dose reduction [32], or referral to physical/occupational therapy was documented [33]. On a case-by-case basis, clinicians were also deemed to have provided appropriate management for moderate-severe CIPN if a recommendation to continue to monitor symptoms was documented or if recommended CIPN management was previously implemented during the study and led to a decrease in CIPN severity. For mild CIPN (PRO-CTCAE™ Numbness and Tingling Severity = 1), clinicians were also found to have provided appropriate management if the presence of CIPN and/or a recommendation to “continue to monitor” was documented. All cases where clinicians did not document CIPN when the patient reported CIPN were rated as inappropriate.

### Procedures

The study consisted of two phases: the usual care phase (UCP) and algorithm phase (AP). All consented clinicians (*N* = 53) participated in both the UCP and AP, but patients (*N* = 162) either participated in the UCP (*n* = 81) or AP (*n* = 81). Patients consented to the UCP completed the CIPN patient-reported outcome measures via iPad in the waiting room prior to seeing the clinician at three consecutive clinic visits associated with neurotoxic chemotherapy (i.e., T1, T2, and T3, respectively). Patients also completed a demographics questionnaire at T1. At T3, clinicians’ CIPN assessment and management documentation and patients’ cancer treatment information were abstracted from the electronic medical record by study staff (T3 must have been completed within a month of neurotoxic chemotherapy completion). Clinicians completed a demographics questionnaire upon enrollment into the study.

**Fig. 1:**
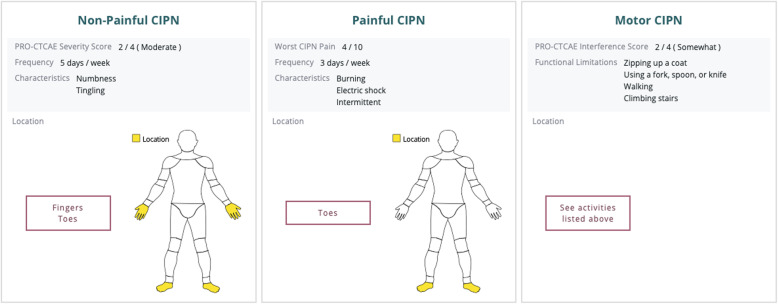
Chemotherapy-Induced Peripheral Neuropathy Symptom Summary Report. The figure describes a sample symptom summary report. The summary report describes the severity, duration, characteristics, and location (i.e., body map) of non-painful and painful CIPN symptoms. In addition, the report describes the degree that CIPN symptoms interfere with activities of daily living

**Fig. 2:**
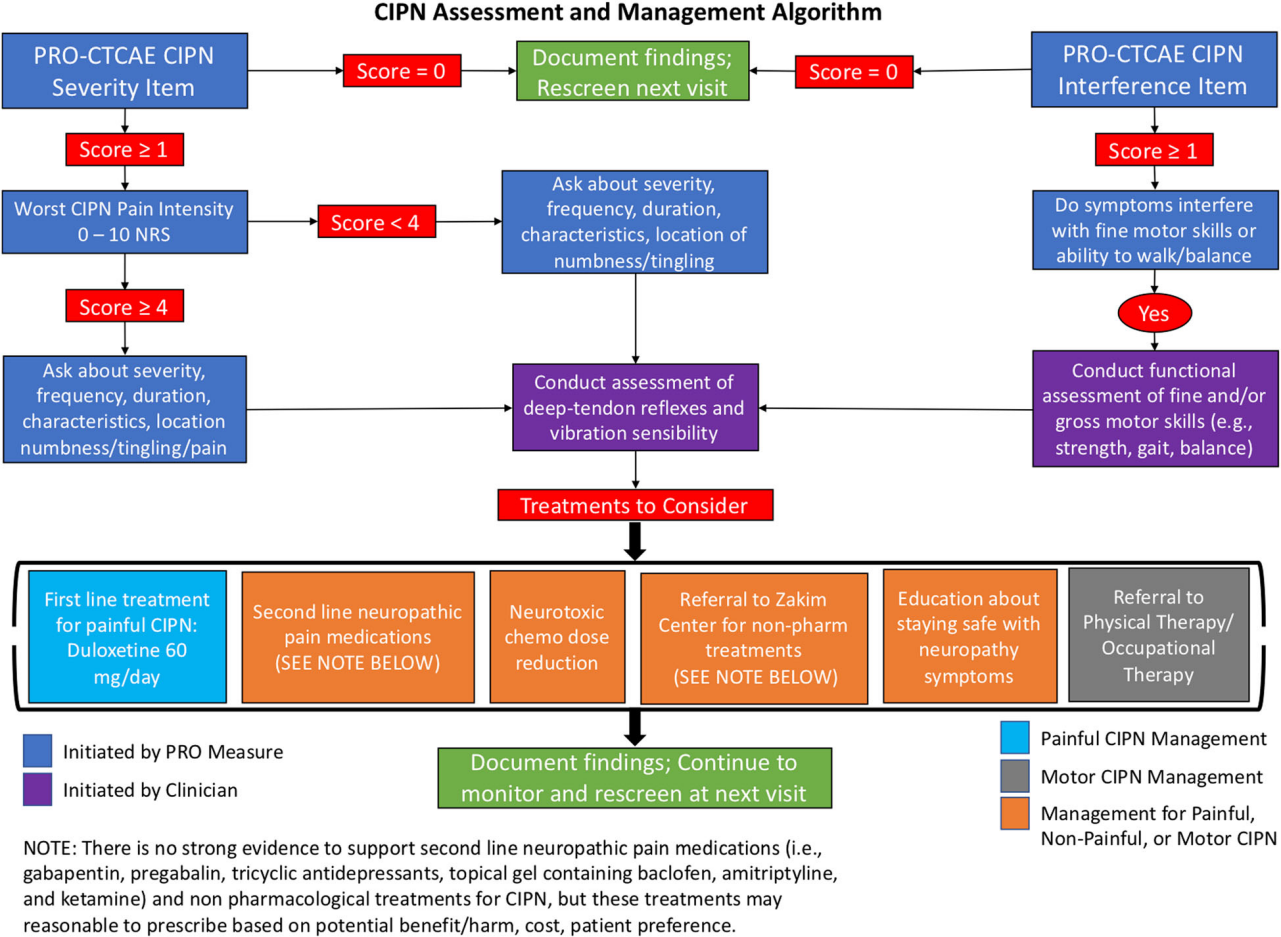
Chemotherapy-Induced Peripheral Neuropathy Assessment and Management Algorithm**.** This figure displays the CIPN assessment and management algorithm that clinicians received during the algorithm phase. The algorithm outlines assessment and management recommendations based on the patients CIPN symptom presentation. Note: The Leonard P. Zakim Center for Integrative Therapies and Healthy Living at Dana-Farber Cancer Institute offers exercise, nutrition, yoga, acupuncture, massage, and mindfulness services.

After the UCP was completed, clinicians received additional CIPN educational materials (i.e., CIPN clinical practice guideline [31], deep-tendon reflexes and vibration sensibility training video, patient-friendly resources about neuropathy safety [34]) and a PowerPoint presentation from the principal investigator about how to use the algorithm (i.e., in-person at faculty meeting or email). During the AP phase, an additional 81 patients were consented. The study procedures were the same as the UCP, except that the survey software generated a color-coded CIPN summary based on the CIPN patient-reported outcome scores (i.e., PRO-CTCAE™ items, 0–10 numerical rating scale of worst CIPN, and follow up questions) and the CIPN algorithm at every clinic visit (i.e., T1, T2, and T3). Study staff printed the CIPN summary (Fig. 1) and algorithm (Fig. 2) and delivered the materials to the clinician (patients received the CIPN summary only) prior to each clinic visit. The CIPN algorithm incorporated recommended CIPN assessment approaches [32, 35] and evidence-based (i.e., duloxetine) [31] or promising management strategies for CIPN [36, 37]. Clinicians were instructed to use the CIPN summary and algorithm for CIPN symptom assessment and management at their discretion. Clinicians completed measures related to feasibility, acceptability, and satisfaction with CIPN algorithm use after the AP was complete.

### Statistical considerations

The primary objectives were to evaluate the impact of the CIPN algorithm on changes in clinicians’ CIPN assessment documentation frequency and adherence to evidence-based CIPN management. The secondary aims were to explore the impact of algorithm implementation on changes in patients’ CIPN symptom severity in comparison to the UCP and to explore clinicians’ perspectives of acceptability and satisfaction with the algorithm. It was estimated that the rate of clinicians’ CIPN assessment documentation and adherence to evidence-based CIPN management would be approximately 30–60% under UCP at T3 [12], and approximately 60% of patients would experience actionable CIPN (e.g., PRO-CTAE™ Numbness and Tingling Severity ≥1/4) at T3. Assuming a 15–30% improvement in the AP, 48 patients (total 96) with actionable CIPN per group, the half-width of a 2-sided 90% confidence interval (CI) for the improvement in clinicians’ CIPN assessment documentation and adherence to evidence-based CIPN management ranged from 0.12 to 0.19.

An index describing clinicians’ frequency of numbness, tingling, and CIPN pain intensity was constructed to evaluate the effect of the algorithm on changes in common CIPN patient-reported outcomes (e.g., if a clinician only documented numbness, the index would be 1/3 = 0.33). The rate of clinicians’ CIPN assessment documentation and adherence to evidence-based management for actionable CIPN (i.e., mild or moderate-severe, respectively) at T3 were compared using Pearson’s chi-squared test for equality of proportions between the UCP and AP.

The mean difference in QLQ-CIPN20 sensory and motor subscale and worst CIPN pain intensity score changes from T1-T3 between the UCP and AP were compared using two sample t-tests. To determine metrics related to feasibility, acceptability, and satisfaction of algorithm implementation, we described the number of times study staff provided the clinicians with the algorithm at each clinic visit and clinicians’ scores on the two-item feasibility questionnaire and Adapted Acceptability E-Scale. All analyses were conducted using data from participants who completed the measures specific to each analysis and no missing data were imputed.

## Results

### Demographic characteristics

**Fig. 3:**
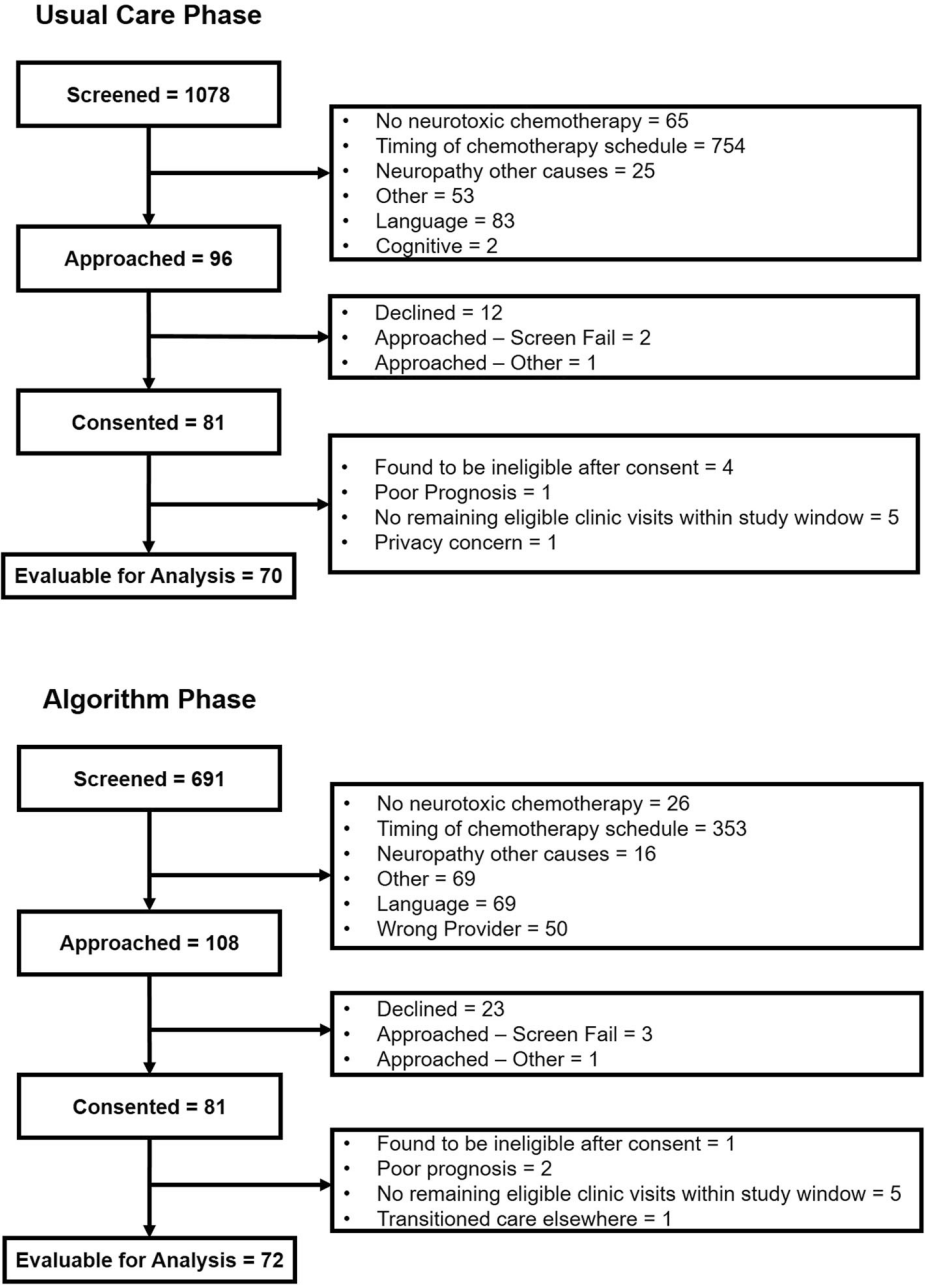
Participant Flow Diagram. This figure describes patients’ progress through the usual care and algorithm phases, respectively

Patient recruitment occurred from June 2018 to November 2019. A total of 162 patients were consented to the study (*n* = 81 UCP; *n* = 81 AP) and 142 patients (*n* = 70 UCP, *n* = 72 AP) were evaluable for the primary or secondary analyses (Fig. 3). Table 1 describes the demographic characteristics of patients who were deemed evaluable for the primary or secondary analyses. Clinician recruitment occurred between 5/15/2018 and 7/26/2019. Of the 88 clinicians invited to participate in the study, 53 consented, 14 declined, and 21 did not respond to our inquiry. The consented clinicians (physician: 54.7%; nurse practitioner: 39.6%; physician assistant: 5.7%) were from the breast (45.3%), gastrointestinal (34%), and multiple myeloma (20.7%) outpatient centers. Clinicians were a median 44 (*Range* = 29–74) years old at the time of consent and majority were female (62.3%) and White (86.8%). T3 notes of 42/53 consented clinicians (79.2%) were reviewed (20 clinicians in both phases, 11 clinicians in the UCP only, and 11 clinicians in the AP only).

**Table 1:** Patient Demographic and Cancer Treatment-related Characteristics

Characteristics	Frequency (***%***)		
	Usual Care (***N*** = 70)	Algorithm (***N*** = 72)	Total (***N*** = 142)
**Age at T1**			
Median (*Range)*	56.5 (30–80)	57.5 (27–79)	57 (27–80)
**Sex**			
Male	21 (30%)	27 (37.5%)	48 (33.8%)
Female	49 (70%)	45 (62.5%)	94 (66.2%)
**Race**			
Asian	3 (4.3%)	3 (4.2%)	6 (4.2%)
White	62 (88.6%)	66 (91.6%)	128 (90.2%)
Black	5 (7.1%)	3 (4.2%)	8 (5.6%)
**Ethnicity (*n*** **= 141)**			
Hispanic	2 (2.9%)	3 (4.2%)	5 (3.5%)
Non-Hispanic White	68 (97.1%)	68 (95.8%)	136 (96.5%)
**Education**			
Did not complete high school	3 (4.3%)	1 (1.4%)	4 (2.8%)
Completed high school	6 (8.6%)	4 (5.6%)	10 (7.1%)
Some college or technical training	20 (28.5%)	15 (20.8%)	35 (24.6%)
University undergraduate training	21 (30%)	26 (36.1%)	47 (33.1%)
University post graduate degree	20 (28.6%)	26 (36.1%)	46 (32.4%)
**Marital Status**			
Single	5 (7.1%)	9 (12.5%)	14 (9.9%)
Married/Partnered	57 (81.5%)	53 (73.6%)	110 (77.5%)
Separated	0	1 (1.4%)	1 (0.7%)
Divorced	5 (7.1%)	3 (4.2%)	8 (5.6%)
Widowed	3 (4.3%)	6 (8.3%)	9 (6.3%)
**Employment Status**			
Working Full-Time	26 (37.1%)	28 (38.9%)	54 (38%)
Working Part-Time	10 (14.3%)	5 (6.9%)	15 (10.6%)
Working at home	1 (1.4%)	1 (1.4%)	2 (1.4%)
Working, but on medical leave	9 (12.9%)	10 (13.9%)	19 (13.4%)
Not working	3 (4.3%)	11 (15.3%)	14 (9.9%)
Retired	21 (30%)	17 (23.6%)	38 (26.7%)
**Computer Use (*n*** **= 139)**			
About once per month	3 (4.3%)	1 (1.4%)	4 (2.9%)
About once per week	4 (5.8%)	2 (2.9%)	6 (4.3%)
More than once per week	62 (89.9%)	67 (95.7%)	129 (92.8%)
**Cancer Type**			
Breast	30 (42.8%)	22 (30.5%)	52 (36.6%)
Gastrointestinal	34 (48.6%)	39 (54.2%)	73 (51.4%)
Multiple Myeloma	4 (5.7%)	11 (15.3%)	15 (10.6%)
Multiple	2 (2.9%)	0	2 (1.4%)
**Cancer Name**			
Breast	30 (42.8%)	22 (30.6%)	52 (36.6%)
Colorectal	16 (22.9%)	25 (34.7%)	41 (28.9%)
Multiple Myeloma	4 (5.7%)	10 (13.9%)	14 (9.9%)
Pancreatic	6 (8.6%)	6 (8.3%)	12 (8.5%)
Esophageal	7 (10%)	3 (4.2%)	10 (7.0%)
Other gastrointestinal^a^	5 (7.1%)	5 (6.9%)	10 (7.0%)
Breast and Colorectal	2 (2.9%)	0	2 (1.4%)
Multiple Myeloma and Breast	0	1 (1.4%)	1 (0.7%)
**Cancer Stage**			
Stage I	10 (14.3%)	10 (13.9%)	20 (14.1%)
Stage II	16 (22.9%)	12 (16.7%)	28 (19.7%)
Stage III	23 (32.8%)	19 (26.4%)	42 (29.6%)
Stage IV Metastatic	12 (17.1%)	21 (29.1%)	33 (23.2%)
Unknown/Not reported	9 (12.9%)	10 (13.9%)	19 (13.4%)
**Chemotherapy Type**			
Taxane	33 (47.1%)	18 (25%)	51 (35.9%)
Platinum	32 (45.7%)	39 (54.1%)	71 (50%)
Bortezomib	2 (2.9%)	3 (4.2%)	5 (3.5%)
Vinca Alkaloid	1 (1.4%)	0	1 (0.7%)
Multiple	2 (2.9%)	12 (16.7%)	14 (9.9%)
**Chemotherapy Name**			
Docetaxel	2 (2.9%)	3 (4.2%)	5 (3.5%)
Docetaxel + Carboplatin	0	1 (1.4%)	1 (0.7%)
Docetaxel + Carboplatin + Abraxane	0	1 (1.4%)	1 (0.7%)
Paclitaxel	31 (44.2%)	15 (20.8%)	46 (32.4%)
Paclitaxel + Carboplatin	0	2 (2.8%)	2 (1.4%)
Oxaliplatin	30 (42.8%)	39 (54.1%)	69 (48.6%)
Cisplatin	2 (2.9%)	0	2 (1.4%)
Bortezomib	2 (2.9%)	3 (4.2%)	5 (3.5%)
Vinorelbine	1 (1.4%)	0	1 (0.7%)
Bortezomib + Lenalidomide	2 (2.9%)	7 (9.7%)	9 (6.4%)
Bortezomib + Pomalidomide	0	1 (1.4%)	1 (0.7%)
**Neurotoxic Chemotherapy Duration at T3**			
Received less than 1/3 of planned treatment	4 (5.7%)	11 (15.3%)	15 (10.6%)
Received at least 1/3 of planned treatment	21 (30%)	27 (37.5%)	48 (33.8%)
Received at least 2/3 of planned treatment	34 (48.6%)	31 (43%)	65 (45.8%)
Completed treatment	11 (15.7%)	3 (4.2%)	14 (9.8%)
**Comorbid Conditions Associated with increased Neuropathy Risk**			
Yes	27 (38.6%)	42 (58.3%)	69 (48.6%)
None^b^	43 (61.4%)	30 (41.7%)	73 (51.4%)
**Previous Neurotoxic Chemotherapy**			
Yes	10 (14.3%)	5 (6.9%)	15 (10.6%)
No	60 (85.7%)	67 (93.1%)	127 (89.4%)
**Neurotoxic Chemotherapy Dose Reduction During Study**			
Yes	20 (28.6%)	27 (37.5%)	47 (33.1%)
No	50 (71.4%)	45 (62.5%)	95 (66.9%)
**Neurotoxic Chemotherapy Dose Reduction Rationale (*n*** **= 47)**			
Abnormal Labs	3 (15%)	5 (18.5%)	8 (17%)
General Poor Tolerability	2 (10%)	2 (7.4%)	4 (8.5%)
Hypersensitivity	0	1 (3.7%)	1 (2.1%)
Multiple Reasons	2 (10%)	3 (11.1%)	5 (10.6%)
Neuropathy-Related	7 (35%)	7 (26%)	14 (30%)
Other Cancer Treatment-Related Symptoms	3 (15%)	4 (14.8%)	7 (14.9%)
Other Health Issues	2 (10%)	3 (11.1%)	5 (10.6%)
Other Plans	0	1 (3.7%)	1 (2.1%)
Progression	0	1 (3.7%)	1 (2.1%)
Unknown	1 (5%)	0	1 (2.1%)

Table 1 describes the demographic and cancer treatment-related symptoms of enrolled patient participants that were evaluable for the primary or secondary analyses

^a^ Other gastrointestinal malignancies included appendix, ampullary, cholangiocarcinoma, gallbladder, gastric, small bowel, cecum

^b^ Comorbid conditions that may have increased chemotherapy-induced peripheral neuropathy risk included anxiety, arthritis, depression, diabetes, fatigue, hearing loss, obesity, other neuropathy, other pain, psychological comorbidity, smoking history

### Primary aim

Overall, numbness, tingling, or pain was documented by clinicians in 110/139 (79.1%) reviewed notes at T3 (Table 2). The CIPN assessment documentation index decreased from 0.6468 (i.e., clinicians evaluated approximately 2/3 CIPN symptoms on average) in the UCP to 0.5440 (i.e., clinicians evaluated approximately less than 2/3 CIPN symptoms on average) in the AP at T3 (*p* = 0.07; 90% *CI:* 0.009, 0.20). Overall, a total of 98 patients reported at least mild or greater CIPN severity at T3. The rate of appropriate CIPN management improved 12.3% from 20/46 (43.5%) patients in the UCP to 29/52 (55.8%) patients in the AP, but the change was not significant (*p =* 0.31, 90% CI: − 0.31, 0.06). The frequency of appropriate mild CIPN management improved 22% from 12/25 (48%) cases in the UCP to 21/30 (70%) cases in the AP, but the change was not significant (*p* = 0.17; 90% *CI:* − 0.47, 0.03). There was no improvement in the frequency of appropriate moderate-severe CIPN management documentation following algorithm implementation (UCP: 8/20 [38.1%]; Algorithm: 8/22 [36.4%]). Duloxetine was never prescribed to any patients reporting moderate-severe CIPN severity in either phase.

**Table 2:** Frequency of Clinicians’ CIPN Assessment and Management Documentation Between the UCP and AP at T3

Assessment Documentation	Frequency (***%***)			
	Usual Care (***N*** = 67)^**f**^		Algorithm (***N*** = 72)	
**Numbness**				
Not Documented	11 (16.4%)		18 (25%)	
Documented	56 (83.6%)		54 (75%)	
**Tingling**				
Not Documented	14 (20.9%)		21 (29.2%)	
Documented	53 (79.1%)		51 (70.8%)	
**CIPN Pain**				
Not Documented	46 (68.7%)		60 (83.3%)	
Documented	21 (31.3%)		12 (16.7%)	
**Reflexes (*n*** **= 114)**				
No Neuropathy Present^a^	19		6	
Not Documented	46 (95.8%)		61 (92.4%)	
Documented	2 (4.2%)		5 (7.6%)	
**Vibration (*n*** **= 109)**				
No Neuropathy Present^a^	20		10	
Not Documented	47 (100%)		62 (100%)	
Documented	0		0	
**Functional Assessment**^b^ **(*n*** **= 114)**				
No Neuropathy Present^a^	20		5	
Not Documented	30 (63.8%)		41 (61.2%)	
Documented	17 (36.2%)		26 (38.8%)	
**Functional Deficits**^c^ **(*n*** **= 109)**				
No Neuropathy Present^a^	20		10	
Not Documented	36 (76.6%)		49 (79%)	
Documented	11 (23.4%)		13 (21%)	
**Appropriate Management of Mild CIPN PRO-CTCAE™ = 1**	**Usual Care**		**Algorithm**	
	**Yes (*n*** **= 12)**	**No (*n*** **= 13)**	**Yes (*n*** **= 21)**	**No (*n*** **= 9)**
Continue to monitor	5 (41.7%)	0	5 (23.8%)	0
Dose Reduction	1 (8.3%)	0	2 (9.5%)	0
No new management offered; provider documented presence of CIPN	6 (50%)	0	14 (66.7%)	0
No documentation of neuropathy	0	8 (61.5%)	0	4 (44.4%)
No new management offered; Discrepancy in severity of provider documented and patient-reported CIPN^d^	0	5 (38.5%)	0	5 (55.6%)
**Appropriate Management of Moderate-Severe CIPN (PRO-CTCAE™ ≥ 2)**	**Usual Care**		**Algorithm**	
	**Yes (*n*** **= 8)**	**No (*n*** **= 13)**	**Yes (*n*** **= 8)**	**No (*n*** **= 14)**
Continue to monitor	0	2 (15.4%)	4 (50%)	0
Dose Reduction	4 (50%)	0	3 (37.5%)	0
No documentation of neuropathy	0	0	0	4 (28.6%)
No new management offered^e^	2 (25%)	11 (84.6%)	1 (12.5%)	10 (71.4%)
Pharmacological Treatment Offered	2 (25%)	0	0	0

Table 2 describes clinicians’ frequency of CIPN assessment documentation and adherence to evidence-based CIPN management at T3 in both the usual care and algorithm phases

^a^If the clinician stated that no neuropathy was present in the reviewed note, we did not code the absence of documentation related to reflexes, vibration, functional assessment, or functional deficits as “Not Documented.” Instead, we removed such instances from the sample size for the variables of reflexes, vibration, functional assessment, or functional deficits. Thus, the frequency of documentation of reflexes, vibration, functional assessment, or functional deficits is calculated from the number of instances in which the clinician documented that neuropathy was present

^b^Included the documentation of gross motor (e.g., walking or balance observations), fine motor (e.g., picking up a penny), or strength tests conducted by the clinician

^c^Included the absence or presence of any limitations in activities of daily living associated with chemotherapy-induced peripheral neuropathy symptoms (e.g., typing, walking, opening jars, writing, weakness, fine motor coordination) documented by the clinician

^d^Patient reported mild neuropathy, but clinician documented that no neuropathy was present

^e^There were three instances where the clinician did not recommend any new CIPN management, but the cases were rated as appropriate CIPN management: 1) the clinician dose reduced neurotoxic chemotherapy prior to T3 and CIPN scores decreased from T2 to T3, 2) the clinician prescribed gabapentin prior to T3 and worst CIPN pain intensity decreased from T2 to T3, and 3) the clinician dose reduced neurotoxic chemotherapy two times prior to T3 and CIPN severity decreased from T1 to T3

^f^*N* = 67 instead of 70 because three patient participants did not see a consented clinician at T3

### Secondary aims

There were no significant changes in patients’ mean QLQ-CIPN20 sensory, QLQ-CIPN20 motor, or worst CIPN pain intensity scores between phases from T1 to T3 (Table 3). The algorithm was successfully delivered to clinicians prior to 207/216 (95.8%) clinic visits. Most clinicians “seldomly” or “sometimes” used the CIPN algorithm and symptom assessment summary to facilitate CIPN symptom assessment and management during a clinic visit, while clinicians “seldomly” used the CIPN education materials study staff members emailed clinicians prior to algorithm use (*n* = 19) (Table 4). Clinicians’ ratings of acceptability and satisfaction were moderate, with individual item score ranges of 1–5 for all eight questions of the Adapted Acceptability E – Scale. The highest rated item was related to how understandable the content was presented within the algorithm (*Mean* = 3.95, *SD* = 1.13), while the lowest rated item pertained to how helpful the algorithm was in guiding CIPN assessment or management clinical interactions with patients (*Mean* = 2.89, *SD* = 1.29) (*n* = 19) (Table 4).

**Table 3:** T1 – T3 Changes in CIPN Patient-Reported Outcomes Between Study Phases

Outcomes	Usual Care ***Mean (SD)***	Algorithm ***Mean (SD)***	Contrast Between Groups^**f**^
**Sensory CIPN Severity**			
T1^a^	8.21 (11.77)	7.48 (9.2)	*t* = 0.06 *p* = 0.95 90% CI = − 2.64, 2.83 *n* = 136
T2^a^	12.62 (12.83)	9.47 (8.74)	
T3^c^	12.68 (12.38)	11.21 (11.45)	
**Motor CIPN Severity**			
T1^a^	5.38 (8.7)	4.93 (6.42)	*t* = 0.12 *p* = 0.90 90% CI = −2.28, 2.64 *n* = 136
T2^b^	7.32 (10.57)	7.89 (8.91)	
T3^c^	8.98 (11.84)	8.04 (9.33)	
**Worst CIPN Pain Intensity**			
T1^d^	1.38 (2.17)	1.43 (1.79)	*t* = 0.18 *p* = 0.86 90% CI = −0.53, 0.65 *n* = 134
T2^e^	2.01 (2.54)	1.97 (2.3)	
T3^c^	2.05 (2.34)	1.90 (2.01)	

Table 3 describes changes in sensory CIPN severity, motor CIPN severity, and worst CIPN pain intensity severity from T1 to T3 between study phases

^a^*N* = 69 Usual Care, *N* = 71 Algorithm

^b^*N* = 69 Usual Care, *N* = 70 Algorithm

^c^*N* = 66 Usual Care, *N* = 72 Algorithm

^d^*N* = 69 Usual Care, *N* = 69 Algorithm

^e^*N* = 68 Usual Care, *N* = 71 Algorithm

^f^Statistical test to determine whether the change from T1 to T3 was different between study phases

**Table 4:** Clinician Acceptability E – Scale and Feasibility Questionnaire Results (*N* = 19)

Adapted Acceptability E – Scale^**a**^	Mean (***SD, Range)***
Did use of the Algorithm help you identify appropriate areas of concern related to the assessment and/or management of CIPN symptoms?	3.05 (1.39, 1–5)
Did use of the Algorithm help guide clinical interactions with patients related to the assessment and management of CIPN symptoms?	2.89 (1.29, 1–5)
Was the Algorithm helpful in promoting communication between you and your patients related to the assessment and management of CIPN symptoms?	3.21 (1.4, 1–5)
Was the Algorithm helpful in identifying areas of need or symptoms related to CIPN?	3.11 (1.15, 1–5)
Was use of the Algorithm helpful in promoting your knowledge related to the assessment and/or management of CIPN symptoms?	2.95 (1.13, 1–5)
How understandable was the content presented within the Algorithm?	3.95 (1.13, 1–5)
How easy was it to use the Algorithm during your clinical interactions with patients? (*n* = 18)	3.39 (1.14, 1–5)
Overall, how would you rate your satisfaction with the Algorithm?	3.11 (0.94, 1–5)
**Feasibility Questionnaire**^**b**^	
When you received the Chemotherapy-Induced Peripheral Neuropathy (CIPN) Symptom Assessment Summary (sheet displaying patients’ CIPN severity scores) and the CIPN Assessment and Management Algorithm, how often did you use the CIPN Symptom Assessment Summary or Assessment and Management Algorithm to aid you in the assessment and management of CIPN during those particular clinic visits?	2.58 (0.9, 1–4)
We sent you several other education materials (i.e., neuropathy safety information, vibration sensibility and deep-tendon reflexes training video, CIPN clinical practice guideline) via email before you began using the CIPN Assessment and Management Algorithm. You most likely received this email around February or March 2019. From the time that you received the email containing the materials, did you review or use the materials we provided to you to aid in the assessment and/or management of CIPN symptoms?	1.89 (0.81, 1–3)

Table 4 describes clinicians’ mean scores on the Adapted Acceptability E – Scale and Feasibility Questionnaire in regard to the CIPN algorithm at the end of the study

^a^The Adapted Acceptability E – Scale items were scored from 1 to 5, with higher scores indicating greater acceptability or satisfaction

^b^The Feasibility Questionnaire items were scored from 1 to 5 (i.e., 1 = Never, 2 = Seldom, 3 = Sometimes, 4 = Frequently, 5 = Always)

## Discussion

Improving CIPN assessment documentation is a two-fold challenge: 1) patients and clinicians must discuss the absence or presence of CIPN and 2) clinicians must document CIPN in the electronic medical record. Results revealed that implementation of the clinician decision support algorithm did not significantly improve clinicians’ documentation of CIPN symptom assessment. The observed CIPN assessment documentation frequency in the UCP (e.g., 83.6 and 79.1% documented numbness and tingling, respectively) was considerably higher than CIPN assessment documentation frequencies reported in prior studies (*Range* = approximately 46 to 58%) [12, 13]. As such, we may have observed a ceiling effect in pre-posttest CIPN assessment documentation changes. On the other hand, clinicians’ painful CIPN documentation in the UCP was low (31.3%), but clinicians’ painful CIPN documentation did not improve following algorithm implementation (16.7%). Improving the identification of painful CIPN is critical as the first-line treatment recommendation of duloxetine for painful CIPN was determined based on evidence suggesting that duloxetine significantly improved average CIPN pain intensity in comparison to placebo [38].

Study results demonstrated that availability of the algorithm did not significantly improve clinicians’ management of mild or moderate-severe CIPN. Although, availability of the algorithm improved clinicians’ management of mild CIPN by 22%, mainly via increased identification and monitoring of CIPN. On the other hand, duloxetine was never prescribed for individuals with moderate-severe CIPN. It has been 7 years since the publication of the trial by Smith et al. (2013) that demonstrated the efficacy of duloxetine for chronic painful CIPN [38]; and 6 years since the publication of the clinical practice guideline suggesting duloxetine as a first-line treatment for painful CIPN [31], however, barriers to duloxetine prescription still persist and should be explored in further research. Our findings are similar with those of Knoerl et al. (2018), who demonstrated that the implementation of an electronic care planning system improved breast oncology nurse practitioners’ management of non-painful CIPN (e.g., mainly via an increase in the number of recommendations to “continue to monitor”), but not painful CIPN (e.g., no recommendation of analgesic such as duloxetine) [12]. Finally, as expected with the minimal improvements in clinicians’ CIPN management behaviors, there were no changes in CIPN severity between patients enrolled to the UCP or AP.

There are several explanations as to why algorithm implementation did not significantly influence clinicians’ CIPN assessment or management behaviors. Despite the high feasibility of algorithm delivery to clinicians, most clinicians “seldomly” or “sometimes” used the algorithm and/or associated educational materials to guide CIPN assessment or management. Clinicians may not have regularly used the algorithm because of a lack of time in the clinic or they found the algorithm-related assessment and management steps burdensome to implement. Moreover, the implementation plan for algorithm delivery was suboptimal. Colquhoun et al. (2017) conducted a systematic review to determine strategies for designing interventions intended to influence clinician healthcare behaviors and concluded that there were four steps critical to designing such interventions: 1) identifying barriers, 2) targeting intervention components to barriers, 3) theory selection, and 4) obtaining clinician feedback [39]. While the algorithm intervention was designed to target barriers identified in prior research [12–14], the current intervention was not guided by implementation theory and clinicians’ perspectives of acceptability and satisfaction with the intervention were not integrated prior to intervention implementation.

Due to the clinicians’ low usage and acceptability and satisfaction ratings of the algorithm in the current study, future research should be directed towards the testing of theory-based implementation interventions to improve the assessment and management of CIPN. Derived from the Expert Recommendations for Implementing Change (identified 73 implementation strategies and associated definitions) [40, 41] and the Conceptual Model of Evidence-Based Practice Implementation [42], the Strategic Implementation Framework recommends varying implementation strategies along the oncology care change process: 1) Setting the Stage (e.g., create guidelines, identify barriers), 2) Active Implementation (e.g., develop educational materials and methods to monitor intervention delivery), and 3) Monitor, Support, Sustain (e.g., provide reminders or coaching) [43]. With the Strategic Implementation Framework in mind, interventions designed to target clinician-related CIPN assessment and management behavior change must incorporate key stakeholders’ feedback (e.g., clinicians) regarding barriers to assessing and managing CIPN early in the intervention design process and provide ongoing feedback and support during intervention implementation [43]. For patients, potential intervention targets may include previously identified enablers and deterrents to patient reporting of CIPN [44]. Factors that enabled patients to report CIPN to clinicians included positive relationships with health care team members, adequate amount of time to discuss CIPN during appointments, potential to talk with the health care team between in-person clinic appointments, and CIPN education prior to treatment [44]. Conversely, factors that deterred patients from reporting CIPN included perceived need to complete the entire chemotherapy regimen, fear of chemotherapy withdrawal due to CIPN, and lack of education surrounding the potential for chronic CIPN [44].

### Limitations

There are several limitations to this research. First, the generalizability of our findings is limited because the patient and clinician samples were homogenous in race and ethnicity and recruited from one academic cancer center. Second, differences in CIPN assessment and management documentation frequency between phases may be influenced by the number of notes written by particular clinicians within each phase (e.g., some clinicians consistently never documented CIPN) or the number of times each clinician received the algorithm during the study (e.g., algorithm exposure or dose). Third, changes in CIPN assessment or management documentation by clinicians may have been a result of external factors to the study due to the lack of a true control group (e.g., clinician-study staff interaction regarding CIPN assessment; increased awareness of CIPN standards of care not related to algorithm use). Fourth, between-group changes in patients’ CIPN severity over time was confounded by the eligibility criteria (e.g., patients were receiving various neurotoxic chemotherapy types/dosages and recruited at different time points during their neurotoxic chemotherapy regimens). Fifth, it is possible that the methods used to measure changes in clinicians’ CIPN assessment and management documentation may not have been sensitive to the unique symptom presentations associated with CIPN due to taxanes, oxaliplatin, or bortezomib. Lastly, the frequency of CIPN assessment and appropriate management observed in this study may not have been representative of actual clinical practice because we did not audio record patient-clinician outpatient encounters and decisions regarding the appropriateness of clinicians’ CIPN management actions at T3 were made based on patients’ self-reported CIPN severity and clinicians’ CIPN documentation only.

## Conclusion

Implementation of a clinician-decision support algorithm did not significantly improve clinicians’ CIPN assessment documentation or use of evidence-based management strategies when providing care to patients receiving neurotoxic chemotherapy at breast, gastrointestinal, or multiple myeloma oncology outpatient centers. Reasons for the lack of algorithm-induced changes in clinicians’ CIPN assessment and management behaviors included that clinicians infrequently used the algorithm or associated educational materials to assess or manage CIPN and a suboptimal intervention implementation plan. Future research should be directed toward the development and testing of theory-guided implementation interventions to improve the assessment and management of CIPN in clinical practice.

## Data Availability

The datasets used and/or analyzed during the current study are available from the corresponding author on reasonable request.

## Abbreviations

AP: Algorithm Phase
CIPN: Chemotherapy-induced peripheral neuropathy
EORTC QLQ C-30: European Organisation for Research and Treatment of Cancer Quality of Life Core Questionnaire 30
PRO-CTCAE™: Patient Reported Outcomes version of the Common Terminology Criteria for Adverse Events
UCP: Usual Care Phase
